# A Dynamic Hysteresis Model and Nonlinear Control System for a Structure-Integrated Piezoelectric Sensor-Actuator

**DOI:** 10.3390/s21010269

**Published:** 2021-01-03

**Authors:** Xiaobiao Shan, Henan Song, Han Cao, Lanshuang Zhang, Xuhang Zhao, Jizhuang Fan

**Affiliations:** State Key Laboratory of Robotics and System, Harbin Institute of Technology, Harbin 150001, China; shanxiaobiao@hit.edu.cn (X.S.); 19B908033@stu.hit.edu.cn (H.S.); Caohan@hit.edu.cn (H.C.); 19S108196@stu.hit.edu.cn (L.Z.); 18S008023@stu.hit.edu.cn (X.Z.)

**Keywords:** sensor-actuator, dynamic hysteresis model, PID control system

## Abstract

The piezoelectric sensor-actuator plays an important role in micro high-precision dynamic systems such as medical robots and micro grippers. These mechanisms need high-precision position control, while the size of the sensor and actuator should be as small as possible. For this paper, we designed and manufactured a structure-integrated piezoelectric sensor-actuator and proposed its PID (Proportion Integral Differential) control system based on the dynamic hysteresis nonlinear model and the inverse model. Through simplifying the structure of the piezoelectric sensor-actuator by the centralized parameter method, this paper establishes its dynamic model and explores the input–output transfer function by taking the relationship between the output force and displacement as the medium. The experiment shows the maximum distance of the hysteresis curve is 0.26 μm. By parsing the hysteresis curve, this paper presents a dynamic hysteresis nonlinear model and its inverse model based on a 0.5 Hz quasi-static model and linear transfer function. Simulation results show that the accuracy of the static model is higher than that of the dynamic model when the frequency is 0.5 Hz, but the compensation accuracy of the dynamic model is obviously better than that of the static model with the increase of the frequency. This paper also proposes a control system for the sensor-actuator by means of the inverse model. The simulation results indicate that the output root mean square error was reduced to one-quarter of the original, which proves that the structure-integrated piezoelectric sensor-actuator and its control system have a great significance for signal sensing and output control of micro high-precision dynamic systems.

## 1. Introduction

The research about piezoelectric sensor-actuators involves the development of composite materials with integrated sensing functions [1], structural health detection and correction [2], flexible actuators with variable stiffness [3,4], nano-level manipulators [5,6], medical robots and micro grippers [7,8]. Piezoelectric sensor-actuators can be divided into two types: structure-integrated and function-integrated. In structure-integrated mode, two devices are utilized to realize the sensing function and actuating function, respectively. However, the function-integrated type takes the sensing function and actuating function as one device, which always leads to the coupling of driving signal and sensing signal. Therefore, the research on function-integrated piezoelectric sensor-actuators is mainly concentrated on how to separate the driving electric signal and sensing electric signal [9]. This paper focusses on the structure-integrated piezoelectric sensor-actuator.

Boukabache et al. [10] studied the application of piezoelectric sensor-actuators in nondestructive testing of aerospace equipment. Zhang et al. [11] utilized the piezoelectric effect to expound a piezoelectric bimorph pump using discrete categories of piezoelectric ceramic as pistons to control the liquid flow while sensing the difference between internal and external pressure. Lee et al. [12] researched the use of adaptive wave cancellation in a new multilayer smart skin sensor which contains piezoelectric materials as actuators and sensors to attenuate the primary low-frequency noise underwater. Varanis et al. [13] discussed the piezoelectric sensor-actuator as an MEMS accelerometer for mechanical vibration analysis while Rathod [14] summarized the applications of piezoelectric sensor and actuators for MENS.

At the same time, some inherent problems of piezoelectric materials have come to people’s attention. Inherent defects such as the creep and hysteresis nonlinear characteristics of piezoelectric ceramics mean the accuracy of high-precision displacement output system cannot be guaranteed, especially in a dynamic system. In some conditions the maximum relative output displacement error is 25% without control [15]. Therefore, many researchers commenced to design operational units to characterize the nonlinearity and establish various hysteresis models in order to analyze and solve the hysteresis problem [16]. For example, Hu [17] demonstrated that the classical Preisach model is efficient enough only when the load fluctuation is relatively small. Mayergoyz [18] proposed a generalized Preisach model while introducing an integral function. Chen et al. [19], Luo et al. [20] and other researchers tried to improve the accuracy of the Preisach model [21]. Meanwhile, Tan et al. [22] essentially derived a singularity free Prandtl–Ishlinskii model from the Preisach model to extend the P-I operator to map the hysteresis data, and developed a feedforward controller. Based on this, there are still many researchers looking to further develop this kind of model, such as Al Janaideh et al. [23,24], Gu et al. [25,26,27] and Hao et al. [28] In addition, Banks et al. [29] derived the Krasonsel’skii–Pokrovskii (K-P) model from the Preisach model. Goldfarb [30] presented a generalized Maxwell resistive capacitor as a lumped parameter causal representation of rate-independent hysteresis. Chen et al. [31] proposed a dynamic model to describe the voltage-force hysteresis curve based on the piezoelectric actuator, as Li et al. [32] did. As for the control system, Ang et al. [33] developed a feedforward controller with an inverse rate-dependent model. Gan et al. [16] thought that feedforward-feedback control and feedback control were the emphasis in the future. Yi et al. [34] reported an adaptive feedforward controller to solve the hysteresis problem. Some other researchers such as Tan et al. [22], Davoodi et al. [35] and Saleem et al. [36] focused on developing feedforward controls. Combining the PID controller with the inverse hysteresis model, the feedforward-feedback control [25] is part of the most popular and effective controls.

However, existing related research on piezoelectric sensor-actuators mainly focuses on how to improve the accuracy of the model to describe the dynamic characteristics of piezoelectric materials. Mostly it focuses on the material properties and the lack of research on the background of structure-integrated piezoelectric sensor-actuators, which means that the research on structural integrated piezoelectric sensor-actuators and their control systems is very scarce. In the existing related research on structural integrated piezoelectric sensor-actuators, the difficulties with the inverse hysteresis model [37] and the poor output accuracy of the actuator [38] are still to be solved. Therefore, based on research on the structure-integrated piezoelectric sensor-actuator, this paper presents a dynamic hysteresis model based on building a nonlinear control system to achieve the displacement correction of the microsystem after sensing, which provided a fine application foreground for a structure-integrated piezoelectric sensor-actuator in a micro high-precision integrated system.

## 2. Design of Piezoelectric Sensor-Actuator

### 2.1. Structure Design of Piezoelectric Sensor-Actuator

**Figure 1:**
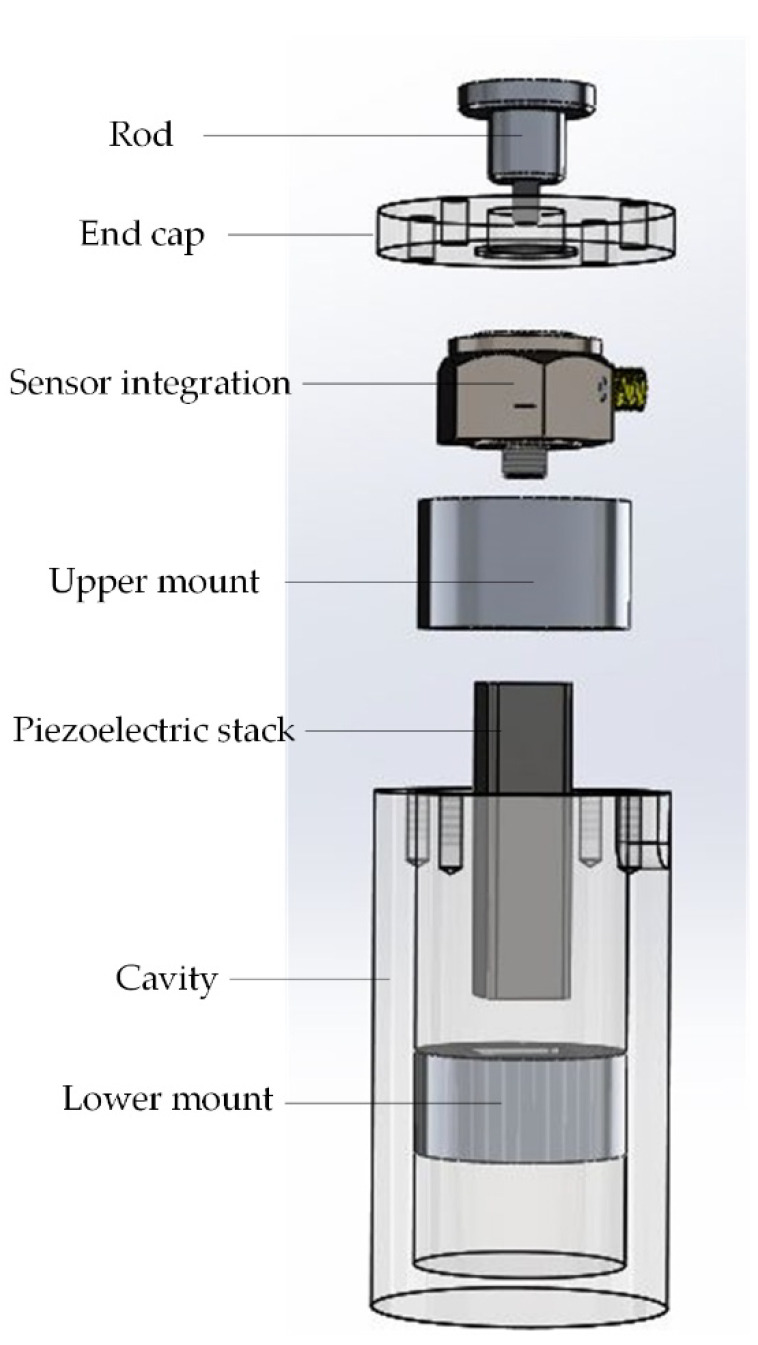
The structure of the piezoelectric sensor-actuator.

**Figure 2:**
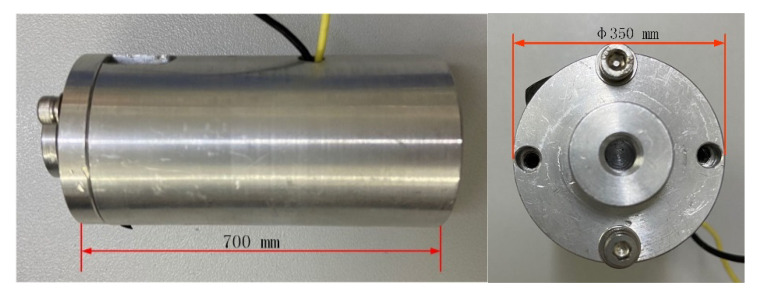
The prototype of the piezoelectric sensor-actuator.

In order to meet the frequency response characteristics, the annular quartz wafer (5 × 12 × 1 mm) was selected and packaged with stainless steel as the sensor part of the system. Considering the load and preload for the structure-integrated sensor-actuator, we employed an electron beam to seal. As for the actuator, the structure of the PZT was 11 × 11 × 37 mm (284 pieces × 0.13 mm). Figure 1 illustrates the structure of the piezoelectric sensor-actuator. The prototype of the piezoelectric sensor-actuator is shown in Figure 2. The parameters of the PZT are listed in Table 1.

### 2.2. The Mathematical Model of the Piezoelectric Sensor-Actuator

The piezoelectric sensor in this paper was an *x*-direction cut quartz wafer, which can produce a tensile piezoelectric effect. The piezoelectric actuator works under the first boundary condition which is that the mechanical end is free and the electrical signal is short-circuited (Specific description is that the piezoelectric stack working under the condition in which stress on the boundary of the piezoelectric stack is zero, but the strain is not zero and the internal electric field is zero or constant, but the potential displacement was not zero or constant). In this way, we calculated the output displacement of the piezoelectric stack as

(1) $$x=S_{3}\cdot n\cdot h=(s_{33}^{E}T_{3}+d_{33}E_{3})\cdot h\cdot n=n\cdot (-\frac{h\cdot s_{33}^{E}}{lb}F_{out}+d_{33}u_{a})$$

where *x* means the displacement of the actuator, *S_3_* means the strain of the piezoelectric stack, *T_3_* means the stress, *d_33_* means the piezoelectric constant, *E_3_* means the external electric field, $S_{33}^{E}$ means the short circuit elastic compliance coefficient with constant electric field, *h* means the thickness of a single piezoelectric ceramic sheet, *n* is the number of layers of piezoelectric laminated ceramics, *l* means the length of the piezoelectric ceramic sheet, *b* means the width of the piezoelectric ceramic sheet, *F_out_* means the direction output force of piezoelectric stack, *u_a_* means the drive voltage of the piezoelectric stack.

This paper describes the piezoelectric driving amplifier as a series circuit of gain *k_amp_* and resistance *R*. Piezoelectric ceramic actuators are described as capacitors *C_a_*. The equivalent physical model of piezoelectric driving amplifier is

(2) $$RC_{a}\frac{du_{a}(t)}{d_{t}}+u_{a}(t)=k_{amp}u_{c}(t)$$

where *u_c_* means the output voltage of driving amplifier.

The equivalent voltage produced by quartz sensor is

(3) $$u_{s}=\frac{q}{C_{s}}=\frac{qh}{\varepsilon_{0}\varepsilon_{r}A}=\frac{d_{11}hF_{out}}{\varepsilon_{0}\varepsilon_{r}A}$$

where *q* means the charge produced by quartz, *A* means the area of the piezoelectric quartz, *h* means the thickness of the quartz, *ε_r_* means the relative permittivity of quartz, *ε*_0_ means the dielectric constant of air.

**Figure 3:**
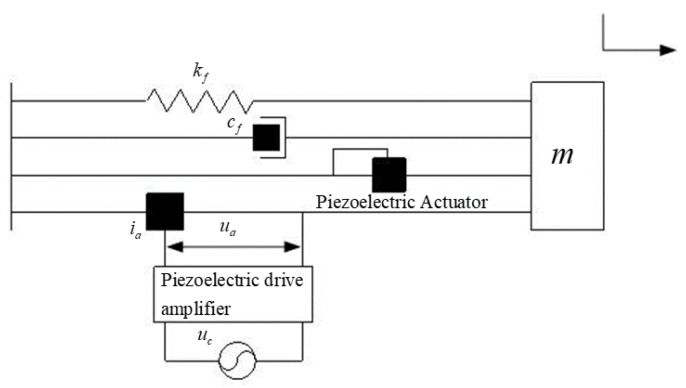
The dynamical model of the piezoelectric sensor-actuator.

Using the centralized parameter method to simplify the system as shown in Figure 3, the system can be equivalent to a spring damping system and the piezoelectric actuator pushes the push rod to carry out one-dimensional reciprocating motion, so that the dynamical model of the system is

(4) $$m\ddot{x}+c_{f}\dot{x}+\left(k_{f}+k_{a}\right)x=F_{out}$$

where *m* is the equivalent mass of moving parts of the piezoelectric sensor-actuator system, *c_f_* is the equivalent viscosity coefficient of actuator structure, *k_f_* is the stiffness coefficient of actuator, *k_a_* means the equivalent stiffness of preload, *F_out_* is the input force of piezoelectric ceramic stack on push rod spring, x is the displacement of push rod end.

Simultaneously, (1)~(4), the differential equation of the system is

(5) $$\begin{array}{l} RC_{a}\frac{du_{a}(t)}{d_{t}}+u_{a}(t)=k_{amp}u_{c}(t)\\ m\ddot{x}+c_{f}\dot{x}+(k_{f}+k_{a})x=k_{a}nd_{33}u_{a}=k_{a}k_{d}u_{a} \end{array}$$

Laplace transforming the above equation, the transfer function from the control voltage generated by the sensor to the displacement output is

(6) $$G_{sys}(s)=\frac{K\omega^{2}}{s^{2}+2\xi \omega_{n}s+\omega_{n}{}^{2}}\cdot \frac{k_{d}k_{amp}}{RC_{a}s+1}$$

### 2.3. Experimental Study on Hysteresis of Piezoelectric Sensor-Actuator

Because of the hysteresis of piezoelectric material, the prototype is checked to explore the relationship between the input and output of piezoelectric sensor-actuator. In this paper, the hysteresis characteristics of the actuator made of piezoelectric ceramic stack are mainly considered. Therefore, the driving voltage generated by the sensor is equal to a sinusoidal excitation signal with 0 ~ 30 V and 0.5 Hz.

**Figure 4:**
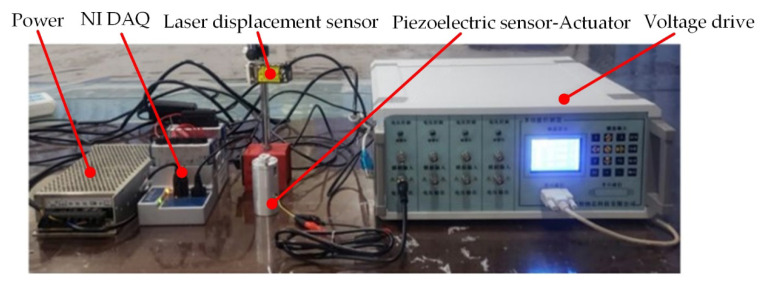
Experimental schematic diagram.

**Figure 5:**
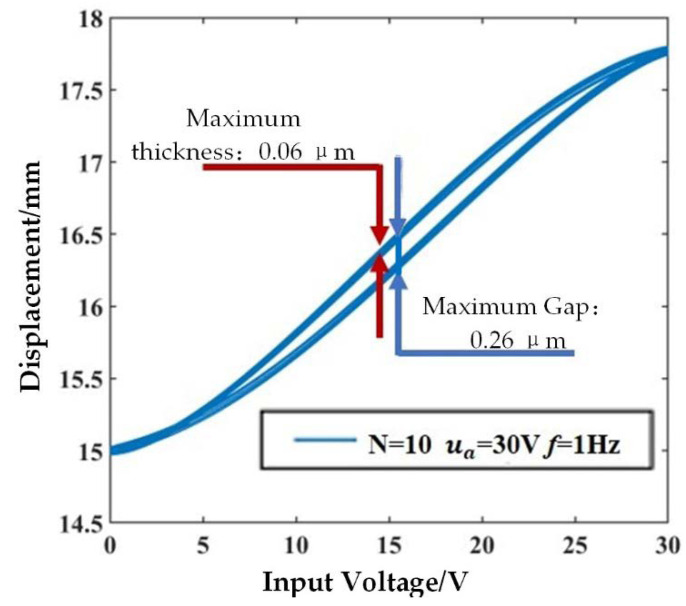
Hysteresis curve of piezoelectric sensor-actuator.

The experiment is shown in Figure 4. Figure 5 illustrates the curve of the end displacement of the push rod with the input voltage signal.

As shown as Figure 5, when the voltage is 15 V, the hysteresis phenomenon is the most obvious, and the distance between the two voltage curves is 0.26 μm. For the same voltage value, the thickest part of the curve is 0.06 μm which means the maximum displacement repetition deviation is 0.06 μm.

## 3. Dynamic Hysteresis Model of Piezoelectric Sensor-Actuator

### 3.1. Dynamic Hysteresis Nonlinear Model of Piezoelectric Sensor-Actuator

**Figure 6:**
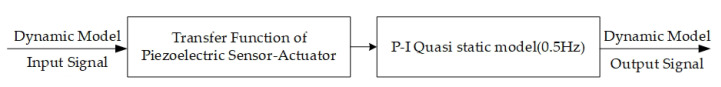
Dynamic Prandtl–Ishlinskii model.

The classical Prandtl–Ishlinskii (P-I) model is a static model which is greatly affected by the value of the play operator and the density function, and it is a linear model. However, as shown in Equation (5), the hysteresis characteristics of piezoelectric actuators with ceramic stacks are closely related to the driving voltage frequency. In order to improve the accuracy of the model, this paper proposed a dynamic model based on the static model. The excitation signal collected by the piezoelectric sensor is input to the 0.5 Hz quasi-static model through the calculation and processing of the transfer function, and then the output signal of the piezoelectric actuator is obtained, as shown in Figure 6.

The modeling accuracy of the classical P-I model is mainly determined by the experimental data acquisition accuracy, the number of operators and the initial value of the density function. In order to obtain a 0.5 Hz quasi-static model, simplify the identification process and shorten the modeling period, the function relationship between the threshold value of independent variable *r* and the density function *q* is simplified to the corresponding relationship with the weight *ω*. 

Therefore, the mathematical expression of the model can be simplified as

(7) $$\Pi [v](t)=qv(t)+\sum_{i=1}^{n}w_{i}F_{r_{i}}[v](t)$$

Nine threshold values are taken as *r*= [0,0.1,0.2,0.3,0.4,0.5,0.6,0.7,0.8]. The identification results are

ω=[0.58751,0.46211,0.06758,0.00092,0.30240,0.99999,0.20197,0.73566,0.15913]. By changing the excitation signal frequency to 0.5, 10, 20 and 50 Hz, the static model and dynamic model of the system are simulated and analyzed. The results are listed in Table 2.

As listed in Table 2, the dynamic model can more accurately describe the dynamic hysteresis characteristics of the piezoelectric actuator after comparing the errors of different models. The relative error of the static model increased with the frequency increasing from 0.5 Hz to 50 Hz. When the frequency is 50 Hz, the average relative error of the static model is four times that of the dynamic hysteresis model. Therefore, the static model can only describe the hysteresis nonlinearity of the piezoelectric sensor-actuator under the input voltage signal near the identification frequency of the reference model for parameter identification. The dynamic model can accurately describe the dynamic hysteresis characteristics of piezoelectric ceramic actuators in a wide frequency range.

### 3.2. Dynamic Hysteresis Nonlinear Inverse Model of Piezoelectric Sensor-Actuator

The dynamic inverse model is designed as a linear inverse model superimposed on the static inverse model. Invert the transfer function $G_{sys}(s)$ and identify the system by results so that

(8) $$G_{inv}(s)=(-42.29s-100400)/(s^{2}+467.8s+27470)$$

From Equation (7), the inverse P-I model is expressed as

(9) $$\Pi^{-1}[v](t)=q^{-1}v(t)+\sum_{i=1}^{n}\hat{p}(r_{i})F_{{\hat{r}}_{i}}[v](t)$$

A mathematical expression of the initial load curve is

(10) $$\varphi (r)=qr+\int_{0}^{r}p(\xi )g(\xi )d\xi \varphi$$

Taking the derivative of Equation (10) and simplifying it while $r\in \left[r_{j},r_{j+1}\right)$, we got

(11) $$\varphi^{\prime }(r)=q+\int_{0}^{r}p(\xi )d(\xi )$$

Threshold of inverse model is ${\hat{r}}_{l}=\varphi \left(r_{l}\right)$, and the recurrence formula is

(12) $${\hat{r}}_{l}={\hat{r}}_{l-1}+\int_{\eta -1}^{\eta }\varphi^{\prime }(r)dr={\hat{r}}_{l-1}+\left(q+\sum_{i=1}^{l-1}p_{i}\right)\left(r_{l}-r_{l-1}\right)$$

Mathematical model of the P-I model and its inverse model is

(13) $$\varphi^{-1^{\prime }}({\hat{r}}_{j})=1/\varphi^{\prime }({\hat{r}}_{j})\;,\;\varphi^{\prime }(r)=q+\sum_{i=1}^{j}p_{i}\;,\;\varphi^{-1^{\prime }}(\hat{r})={\hat{q}}^{-1}+\sum_{i=1}^{j}{\hat{p}}_{i}$$

When $\left[r_{j},r_{j+1}\right)$, simultaneous Equations (9)–(13), we got

(14) $${\hat{q}}^{-1}+\sum_{i=1}^{j}{\hat{p}}_{i}=1/(q+\sum_{i=1}^{j}p_{i}).$$

While j=1,

(15) $${\hat{q}}^{-1}+{\hat{p}}_{1}=1/(q+p_{1})\;\mathrm{means}\;{\hat{p}}_{1}{=-p}_{1}/\left[(q(q+p_{1}\right]$$

While j=2,

(16) $${\hat{q}}^{-1}+{\hat{p}}_{1}+{\hat{p}}_{2}=1/(q+p_{1}+p_{2})\;\mathrm{means}\;{\hat{p}}_{2}{=-p}_{2}/{[(q+p}_{1}{+p}_{2}{)(q+p}_{1})]$$

While j=n,

(17) $${\hat{q}}^{-1}+{\hat{p}}_{1}+{\hat{p}}_{2}+\ldots +{\hat{p}}_{n}=1/(q+p_{1}+p_{2}+\ldots +p_{n})\;\mathrm{means}\;{\hat{p}}_{n}=-p_{n}/[q+\sum_{i=1}^{n}p_{i}(q+\sum_{i=1}^{n-1}p_{i})]$$

The mathematical model of the P-I inverse model is

(18) $$q^{-1}=1/q\;,\;{\hat{r}}_{j}=qr_{j}+\sum_{i=1}^{j-1}p_{i}(r_{j}-r_{i})\;,\;{\hat{p}}_{j}=-p_{j}/[q+\sum_{i=1}^{j}p_{i}(q+\sum_{i=1}^{j-1}p_{i})]$$

Based on the threshold $r^{\prime }=\left[0,0.0588,0.1637,0.2619,0.3600,0.5167,0.5653,0.4884,0.687\right]$ and the initial value $y_{0}{}^{\prime }=[0.5000,0.5587,0.6637,0.7015,0.6034,0.4750,0.4467,0.3981,0.2760]$, identification results of  w ′ are 

**Figure 7:**
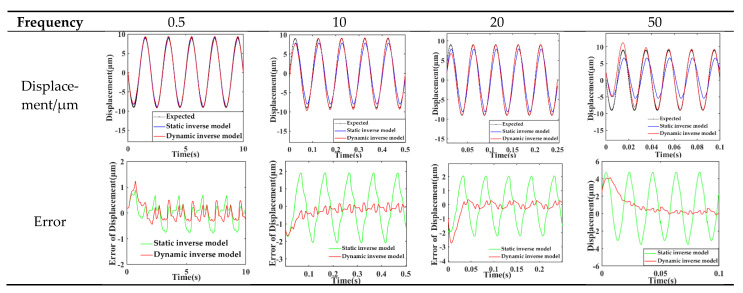
The error between output displacement and ideal output displacement.

ω=[1.70209,−0.74937,0.06556,0.00096,−0.24013,2.74803,−1.46737,−1.24088,−0.09441]. This paper shows the results of the simulation according to the identification result in Figure 7 and lists the errors in Table 3.

Figure 7 shows that when the frequency is 0.5 Hz, the output displacement curve of the static hysteresis inverse model feedforward compensation had a minute distortion within 1 μm and a phase deviation. However, the input–output curve based on the dynamic model almost coincided and the error is smaller than that of the static inverse model except for the first period. With the increase of frequency, the output displacement curve of the static hysteresis inverse model showed obvious distortion and phase deviation but the dynamic model still coincided and the error is significantly reduced. When the frequency reached 50 Hz, there is a large overshoot in the first cycle even though the input and output curves of the dynamic model are basically coincident and the peak displacement is properly compensated.

It can be seen from Table 3 that compared with the static inverse model, the output displacement error of the piezoelectric actuator under the feed-forward compensation of the dynamic inverse model is smaller. With the increase of input voltage frequency, the maximum error, average error and root mean square error of the stationary inverse model after pure adjustment show an upward trend. The maximum error and root mean square error of the output displacement of the dynamic inverse model after pure adjustment are approximate, but the average error is greatly reduced and the fluctuation is small, which is about ¼ ~¹/_3_ that of the static model. The static inverse model is only suitable for improving the hysteresis nonlinearity of the piezoelectric sensor-actuator under quasi-static condition, and the dynamic inverse model has better performance in a wide frequency band.

## 4. PID Control System of the Piezoelectric Sensor-Actuator

**Figure 8:**
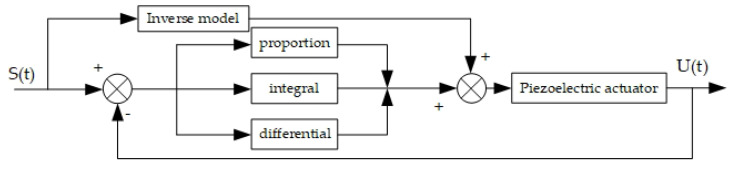
Control system structure with inverse model feedforward and feedback.

In order to make the output displacement more accurate and stable, it is necessary to reduce the displacement overshoot in the first cycle. The design used the inverse model feedforward and feedback as the control system, as shown in Figure 8.

**Figure 9:**
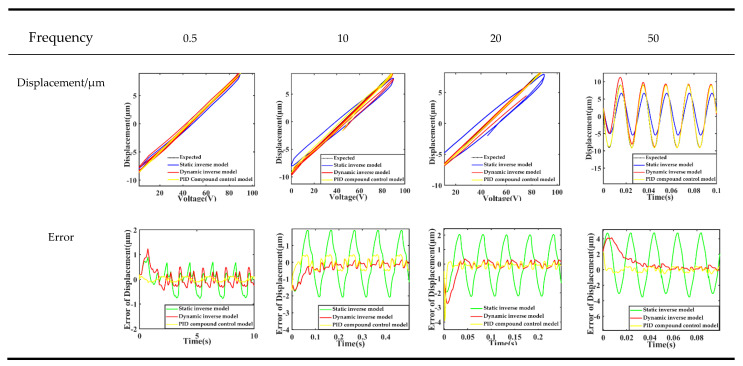
The results of displacement of different control systems.

The design amplitude is 45 V, the frequency is 0.5, 10, 20 and 50 Hz. Sinusoidal AC signals are the excitation signal; the piezoelectric sensor-actuator with PID control system is simulated. The results are shown in Figure 9, and the calculated output displacement errors are listed in Table 4.

As shown in Figure 9, the output displacement hysteresis curve is narrower, the peak value is closer to the ideal value, and the overall curve is more suitable for the ideal linear relationship between input voltage and output displacement. Compared with the other two control schemes which only applied inverse model feedforward control, the error of the output displacement curve entered the stable range more quickly.

It can be seen from Table 4 that the maximum error is not significantly improved under the integrated scheme of feedforward and feedback control of hysteresis inverse model; for example, the output displacement error of inverse dynamic inverse model feedforward control even appeared at 20 Hz, but its average error and root mean square error are effectively improved within the tested frequency range. When the input frequency is 0.5 Hz, the average error is reduced from 0.270 to 0.071, and the root mean square error is reduced from 0.337 to 0.079, which is about 1/4 of the original. When the input frequency is 50 Hz, the average error is reduced from 0.464 to 0.133, and the root mean square error is reduced from 1.052 to 0.250, about ¹/_3_ and ¼ of the original, respectively.

## 5. Result and Discussion

For this paper, a structure-integrated piezoelectric sensor-actuator and its control system are designed and built. The static model of the output displacement is derived theoretically and the stationary model parameters and the dynamic model are calculated by the system identification. This paper proposed the input–output transfer function. Comparing the different models, we found that it can be concluded from the simulation as follows: 

(1) When the frequency increased from 0.5 Hz to 50 Hz, the average fitting error of static hysteresis model increased from 0.027 to 0.187, and for the dynamic hysteresis model from 0.044 to 0.052. The increase for the static inverse model is from 0.316 to 1.248, and for the dynamic inverse model is from 0.27 to 0.464. The results of the average fitting errors showed that the fitting accuracy of the static model is slightly higher than that of the dynamic model at low frequency (0.5 Hz).

(2) The compensation accuracy of the dynamic model is obviously better than that of the static model with the increase of frequency, and the fitting accuracy of the dynamic inverse model is always better than that of the static inverse model in the range of 0.5 to 50 Hz. 

(3) Based on the dynamic inverse model, the average error of piezoelectric sensor-actuator is reduced from 0.270 to 0.071, and the root mean square error is reduced from 0.337 to 0.079 when the input frequency is 0.5 Hz. The root mean square error of the system output displacement is reduced from 1.052 to 0.250, which is about ¼ of the original. This shows that the piezoelectric sensor-actuator control system designed for this paper can basically realize the input–output control of the sensor-actuator.

In summary, the accuracy of the static model is greater than that of the dynamic model when the frequency is 0.5 Hz but the case is obviously opposite beyond 0.5 Hz. This is consistent with the discussion in the theoretical part. However, for the inverse model, the accuracy of the dynamic model is always higher than that of the static model, which shows that the hysteresis characteristics of piezoelectric stacks are nonlinear and are greatly affected by the frequency. In the application background of industrial machinery, the frequency is usually greater than 0.5 Hz. Therefore, comparing with the static model, the dynamic model proposed in this paper obviously has higher accuracy and wider applicable bandwidth.

This structure-integrated sensor-actuator makes full use of the characteristics of piezoelectric materials such as fast response rate. Meanwhile, it can collect deformation signals and control the output of the actuator in real time. This means that we can improve the response rate and accuracy of the system and solve the problem of interference between two signals for function-integrated sensor-actuators.

## 6. Conclusions

As shown in our results, the dynamic model can improve the accuracy of sensor-actuators with the increase of frequency and broaden the available bandwidth of the systems. When combined with the PID control presented in this paper, the root mean square error of the system output displacement is reduced by about one-quarter of the original, which means that the structure-integrated piezoelectric sensor-actuator and its control system could be applied to micro high-precision integrated systems such as medical manipulators and end-effectors for industrial robots. The structure implemented solved the sensing and actuating function problems at the same time.

## Figures and Tables

**Table 1:** The parameters of the PZT.

Component	Material	Elastic Modulus (GPa)	Poisson Ratio	Density *ρ* (kg/m^3^)
Piezoelectric stack	PZT-554	—	0.34	7600
Quartz wafer	*x*-quartz	—	0.17	2650
Sensor housing	steel	206	0.25	7900
Package cavity	aluminum	72	0.33	2810
Fixed seat	aluminum	72	0.33	2810
Thrust bar	aluminum	72	0.33	2810

**Table 2:** Error of hysteresis model under different excitation signal frequencies.

	Frequency of Input Signal/Hz.	0.5	10	20	50
Errors					
Maximum error of static model		0.06529	0.24479	0.27672	0.29893
Average error of static model		0.02707	0.07615	0.09984	0.18748
Root mean square error of static model		0.03394	0.08634	0.11256	0.20978
Maximum error of dynamic model		0.12678	0.17687	0.15577	0.78268
Average error of dynamic model		0.04434	0.04614	0.04096	0.05255
Root mean square error of dynamic model		0.05940	0.05288	0.04746	0.08927

**Table 3:** Errors of the hysteresis inverse model under different excitation signal frequencies.

	Frequency of Input Signal/Hz.	0.5	10	20	50
Errors					
Maximum error of static inverse model		0.90116	2.08209	2.24139	4.76271
Average error of static inverse model		0.31638	0.28382	0.30930	1.24777
Root mean square error of static inverse model		0.40279	0.63755	0.70053	1.99596
Maximum error of dynamic inverse model		1.23827	1.68336	2.73286	4.13814
Average error of dynamic inverse model		0.27003	0.08209	0.08725	0.46418
Root mean square error of dynamic inverse model		0.33745	0.25077	0.33672	1.05211

**Table 4:** The calculated output displacement error of different control systems.

Input Signal Frequency /Hz	0.5	10	20	50
Maximum error	0.20094	1.68336	3.92499	2.28057
Averahe error	0.07136	0.07545	0.05028	0.13326
Root mean square error	0.07998	0.17840	0.22415	0.24960

## Data Availability

Not applicable.

## Nomenclature

*x*	the displacement of the actuator
*S*_3_	the strain of the piezoelectric stack
*T*_3_	stress
*d*_33_	piezoelectric constant
*E*_3_	external electric field
$S_{33}^{E}$	short circuit elastic compliance coefficient with constant electric field
*h*	the thickness of a single piezoelectric ceramic sheet
*n*	the number of layers of piezoelectric laminated ceramics
*l*	the length of the piezoelectric ceramic sheet
*b*	the width of the piezoelectric ceramic sheet
*F*_out_	the direction output force of piezoelectric stack
*u_a_*	the drive voltage of the piezoelectric stack
*k_amp_*	series circuit of gain
*R*	resistance
*C_a_*	capacitors
*u_c_*	the output voltage of driving amplifier
*q*	the charge produced by quartz
*A*	the area of the piezoelectric quartz
*ε_r_*	the relative permittivity of quartz
*ε*_0_	the dielectric constant of air
*m*	the equivalent mass of moving part of piezoelectric sensor-actuator system
*c_f_*	the equivalent viscosity coefficient of actuator structure
*k_f_*	the stiffness coefficient of actuator
*k_a_*	the equivalent stiffness of preload
